# Effect of New Zealand Sujon blackcurrant on resting cardiovascular function in triathletes

**DOI:** 10.1186/1550-2783-11-S1-P3

**Published:** 2014-12-01

**Authors:** Mark ET Willems, Stephen D Myers, Matthew D Cook, Mandy L Gault

**Affiliations:** 1 Department of Sport and Exercise Sciences, University of Chichester, Chichester, United Kingdom

## Background

Blackcurrant contains anthocyanin, a component known to induce vasorelaxation and vasodilation in rat aortic rings [1]. In humans, blackcurrant intake has been reported to increase peripheral blood flow [2], with higher anthocyanin intake having beneficial effects on cardiovascular function in women [3]. However, the effect of blackcurrant intake on cardiovascular function in endurance-trained athletes is unknown. We examined the effect of 1-week Sujon blackcurrant powder supplementation on resting cardiovascular function of trained triathletes.

## Methods

Thirteen healthy triathletes with >3 years experience (8 men; mean±SD: age: 38±8 years, height: 174±5 cm, body mass: 71±9 kg, BMI: 23±2, BF%: 19±5%, VO_2_max: 49.1±6.2 mL kg^-1^ min^-1^, maximum power: 305±68 W) volunteered. Participants were tested following 7 days of Sujon blackcurrant powder (S, 6g/day) or placebo (P) intake, administered following a double-blind, crossover, randomized design with a wash-out period of 4 weeks. Cardiovascular function was recorded for 20 min in supine participants using a beat-to-beat blood pressure monitoring system (Portapres® Model 2, Finapres Medical Systems BV, Amsterdam, The Netherlands). Cardiovascular measures were averaged over 10 consecutive beats, with the lowest systolic blood pressure (BP) and associated measures analysed. Paired two-tailed t-tests were used for analysis with significance accepted at p≤.05. Consent to publish the results was obtained from all participants.

## Results

There were no differences in systolic BP (P: 121±23, S: 120±23 mmHg, p=.92), diastolic BP (P: 69±16, S: 63±14 mmHg, p=.12), mean arterial BP (P: 86±18, S: 82±18 mmHg, p=.33), and heart rate (P: 58±9, S: 59±10 beats min^-1^, p=.95). Stroke volume (P: 82±23, S: 99±25 mL, p<.01) and cardiac output (P: 4.8±1.6, S: 5.8±1.7 L, p<.05) were increased by 25% and 26%, respectively. There was a 16% lower total peripheral resistance (P: 20.2±8.9, S: 15.2±5.3 mmHg L^-1^ min^-1^, p=.05). The changes in resting cardiovascular function were observed in 10 participants.

## Conclusions

Resting cardiovascular function of trained endurance athletes responds positively to 1-week intake of New Zealand Sujon blackcurrant powder. Intake of New Zealand Sujon blackcurrant powder is associated with 1) an increase in stroke volume and cardiac output, and 2) a decrease in total peripheral resistance. For resting skeletal muscles, these observations may influence the delivery of nutrients and clearance of metabolites. The effect on New Zealand Sujon blackcurrant on resting cardiovascular function may support the recovery of endurance athletes.

## References

[B1] Ziberna L Kim JH Auger C Passamonti S Schini-Kerth V Role of endothelial cell membrane transport in red wine polyphenols-induced coronary vasorelaxation: involvement of bilitranslocase Food Funct 2013 4 10 1452 6 10.1039/c3fo60160a 23963285

[B2] Matsumoto H Kamm KE Stull JT Azuma H Delphinidin-3-rutinoside relaxes the bovine ciliary smooth muscle through activation of ETB receptor and NO/cGMP pathway Exp Eye Res 2005 80 3 313 22 10.1016/j.exer.2004.10.002 15721614

[B3] Jennings A Welch AA Fairweather-Tait SJ Higher anthocyanin intake is associated with lower arterial stiffness and central blood pressure in women Am J Clin Nutr 2012 96 4 781 8 10.3945/ajcn.112.042036 22914551

